# Cyclopædia of the Practice of Medicine

**Published:** 1875-05-15

**Authors:** 


CYCLOPÆDIA OF THE PRACTICE OF MEDICINE. Edited by H. von Ziemssen, Professor of Clinical Medicine in Munich, Bavaria. Vol. II.

After a little more delay than was anticipated, the second volume of this important work has made its appearance. In faithfulness of translation, and in typographical appearance, it fully sustains the character of the first volume, and is worthy of commendation. Like the first volume, it is devoted to the consideration of "Acute Infectious Diseases." Varicella, measles, rubeola, and scarlet fever are treated by Prof. Louis Thomas, of Leipzig; small-pox by Dr. Curschmann, of Berlin; erysipelas, miliary fever, dengue, influenza, and hay fever, by Dr. Zuelzer, of Berlin; malarial diseases by Prof. Hertz, of Amsterdam; epidemic cerebro-spinal meningitis by Prof. von Ziemssen, of Munich.

The reader will see that these subjects are among the most important that engage the attention of the practitioner, and they have received a very full and interesting consideration by the several authors. In the departments of history, ætiology, pathology and diagnosis, the work is particularly full and valuable.

In treatment we find nothing new; and yet the general summary in regard to the management of each disease is remarkably judicious, and free alike from the therapeutic skepticism that leads to doing nothing, and the theoretical zeal that demands over-medication.

If we felt disposed to criticize any part of this volume, it would be that relating to the treatment of epidemic cerebro-spinal meningitis.

We think Prof. von Ziemssen has not quite done full justice to this part of the subject. But our object is merely to call the attention of our readers to the progress in the publication of this most valuable work.

Every practitioner who can afford the money will find this Cyclopædia of the Practice of Medicine one of the most valuable additions he can make to his library. The American edition is published by William Wood and Company, 27 Great Jones street, New York. N. S. D.

